# Tutor assessment of PBL process: does tutor variability affect objectivity and reliability?

**DOI:** 10.1186/s12909-019-1508-z

**Published:** 2019-03-08

**Authors:** Bidyadhar Sa, Chidum Ezenwaka, Keerti Singh, Sehlule Vuma, Md. Anwarul Azim Majumder

**Affiliations:** 1 Centre for Medical Sciences Education, The Faculty of Medical Sciences, The University of the West Indies, St Augustine Campus, St Augustine, Trinidad and Tobago; 2 Department of Para-clinical Sciences, Faculty of Medical Sciences, The University of the West Indies, St Augustine Campus, St Augustine, Trinidad and Tobago; 3 Faculty of Medical Sciences, The University of the West Indies, Cave Hill Campus, Bridgetown, Barbados

**Keywords:** Problem based learning, Process Assessment, Tutor variability, Objectivity, Reliability

## Abstract

**Background:**

Ensuring objectivity and maintaining reliability are necessary in order to consider any form of assessment valid. Evaluation of students in Problem-Based Learning (PBL) tutorials by the tutors has drawn the attention of critiques citing many challenges and limitations. The aim of this study was to determine the extent of tutor variability in assessing the PBL process in the Faculty of Medical Sciences, The University of the West Indies, St Augustine Campus, Trinidad and Tobago.

**Method:**

All 181 students of year 3 MBBS were assigned randomly to 14 PBL groups. Out of 18 tutors, 12 had an opportunity to assess three groups: one assessed 2 groups and 4 tutors assessed one group each; at the end each group had been assessed three times by different tutors. The tutors used a PBL assessment rating scale of 12 different criteria on a six-point scale to assess each PBL Group. To test the stated hypotheses, independent t-test, one-way ANOVA followed by post-hoc Bonferroni test, Intra Class Correlation, and Pearson product moment correlations were performed.

**Result:**

The analysis revealed significant differences between the highest- and lowest-rated groups (t-ratio = 12.64; *p* < 0.05) and between the most lenient and most stringent raters (t-ratio = 27.96; *p* < 0.05). ANOVA and post-hoc analysis for highest and lowest rated groups revealed that lenient- and stringent-raters significantly contribute (*p* < 0.01) in diluting the score in their respective category. The intra class correlations (ICC) among rating of different tutors for different groups showed low agreement among various ratings except three groups (Groups 6, 8 and 13) (*r* = 0.40). The correlation between tutors’ PBL experiences and their mean ratings was found to be moderately significant (*r* = 0.52; *p* > 0.05).

**Conclusion:**

Leniency and stringency factors amongst raters affect objectivity and reliability to a great extent as is evident from the present study. Thus, more rigorous training in the areas of principles of assessment for the tutors are recommended. Moreover, putting that knowledge into practice to overcome the leniency and stringency factors is essential.

## Background

Problem based learning (PBL) is adopted by many medical schools worldwide. PBL approach rests the responsibility of learning on students [1, 2]. This problem-solving approach encourages them to take center stage in case-based, self-directed learning and explore the pool of knowledge from varied sources using an active learning process to realize their learning objectives [2]. Since its introduction more than four decades ago, PBL is found to be more active and engaging learning than the traditional approaches of teaching [1–4] - it helps to promote critical thinking in students, sharpen their communication skills, enhance general professionalism, increase retention knowledge and transferable skills, and develop teamwork and collaborative skills [3–5]. It discourages students from rote memorization and simple acquisition of knowledge but encourages and emphasizes the integration of basic knowledge and clinical skills [4–6]. However, the major challenge for PBL is in the assessment of its process. In PBL, tutors’ role is different from the role of a teacher in a traditional and didactic teaching setting [7]. Tutors facilitate active learning, encourage critical thinking, and promote self-directed learning among students [3–5]. The tutors’ role is described as ‘conducive’ or ‘facilitative’ [8] which requires understanding of the learning process [9]. Both (?) tutor and tutoring are important factors which influence PBL process and learning outcomes [10]. Though tutors are in a better position to assess students’ skills and abilities during the PBL process, several studies highlighted the difficulty in generating reliable ratings of the tutors [11–14]. The outcome of tutors’ evaluation of students in PBL tutorials has been contentious in terms of the validity of the ratings and scores given to different students [10–14]. Similar ‘hawk-dove’ effect has been observed in clinical examination where examiners differ in their relative leniency or stringency [15]. Hawks usually fail more candidates, whereas doves tend to pass most candidates [15]. Rater variability in student assessments is found to be problematic in medical education [16] and harsh or inconsistent rater can pose negative consequences for students’ outcome [17]. The literature review showed that ‘hawk-dove’ phenomenon was not extensively studied in problem-based learning. This may be due to the absence of an ‘effective statistical technique’ to examine it [15]. Well trained tutors using well-constructed rubrics may eliminate these discrepancies [11–13, 18].

In order to generate reliable ratings in PBL, Ingchatcharoena et al. (2016) recommended developing rater context factors consisting of rater’s motivation, accountability, conscientiousness, rater goals and ability for rating’ [19]. Mook et al. (2007) identified factors limiting the assessment of students’ professional behavior in PBL which includes absence of effective interaction, lack of thoroughness, tutors’ failure to confront students with unprofessional behavior, lack of effort to find solutions and lack of student motivation [20]. Dolmans et al. (2006) tried to explore the relationship between grades of students’ professional behavior and students rating of tutor performance in PBL and found that tutor performance ratings were not significantly related to harshness of students’ grading. However, the explanations supplemented by authors was two-fold i.e. tutors’ performance ratings were based on rating by groups of students; the percentage of tutors who rated students’ professional behavior as unsatisfactory, was low [21]. Therefore, it is difficult to deny that ratings reflect tutors’ leniency or harshness in judging professional behavior rather than their real contribution to student learning. This phenomenon is referred to as the ‘grading leniency effect’ – students may give higher than deserved rating to the tutors if they received higher than deserved grades [21]. The opposite of leniency effect is the harshness effect; i.e. low grading teachers may receive lower than deserved ratings [22–25]. Indeed, it has been reported that examiners differ significantly in their degree of severity and this might reflect in PBL tutors’ assessment [15, 20, 26].

Although tutorial assessment in PBL is thought to be a valid approach on the learning process, research reports have shown that facilitator assessment can be unreliable [27]. Indeed, human factors such as personal bias, errors/effects such as leniency effect, stringency effect, central tendency error, logical error, and halo effect may affect tutors’ rating of students in PBL [3]. The aim of this study was to determine the extent of tutor variability in assessing the PBL process in the School of Medicine, The University of the West Indies (UWI), St Augustine Campus, Trinidad.

## Methods

The medical school at the UWI, St Augustine Campus, Trinidad, uses a hybrid system of PBL and lectures/laboratory practicals since its inception in 1989 [7, 28]. The school follows the seven-step systematic approach of PBL developed by the University of Limburg, Maastricht [29]. A PBL group, which meets once a week, comprises 11–13 students and a tutor and all used the same PBL cases.

The study population were all tutors (*n* = 18) involved in the facilitation of 3rd year Bachelor of Medicine and Bachelor of Surgery (MBBS) students. All 181 students were assigned randomly to 14 groups. In this study, each tutor was described with the letter T (T1-T18) and each class Groups with a letter G (G1-G14). Out of 18 tutors, 12 had the opportunity to assess three groups, one assessed 2 groups and 4 tutors assessed one group each. At the end each group was assessed three times by different tutors using the PBL assessment rubrics as mentioned below.

All students were familiar with the PBL process as they received formal orientation regarding PBL at the beginning of the Year 1. It is the university-established policy that all tutors received necessary structured training in PBL delivery and assessment. The structured training covers topics such as, an introduction to the educational philosophy of PBL, systematic approach to PBL, the role of the tutor as a facilitator, encouraging critical thinking and self-directed learning, PBL process assessment and rubrics.

The tutors were required to rate each student on his/her involvement and contribution in the PBL process in solving PBL cases utilizing the Maastricht seven-step approach [29]. For the student rating, tutors used the University of the West Indies PBL tutorial assessment rating scale [30]. The rating scale consists of 13 items covering 12 performance criteria and one global assessment which were to be rated on a six-point scale (Very Poor (0), Poor (1), Adequate (2), Good (3), Very Good (4) and Excellent (5). The first 12 criteria included: (i) Ability to clarify, define and analyze problem; (ii) ability to generate and test hypotheses; (iii) ability to generate learning objectives; (iv) ability to select, sort, synthesize & evaluate learning resources; (v) cognitive reasoning/critical thinking skills; (vi) self-monitoring skills; (vii) demonstrating initiative, curiosity and open-mindedness; (viii) organization and preparation for group sessions; (ix) commitment and participation in group sessions; (x) ability to express ideas & use language; and (xi) collaborative decision making skills; and (xii) team skills. In the last item, tutors used the six-point rating scale as Novice (0), Beginning (1), Developing (2), Accomplished (3), Exemplary (4), Master (5) to assess the global performance/competence of the student. On this scale, “novice” indicated below basic competence, “beginning” and “developing” students indicate having achieved basic competence, “accomplished” and “exemplary” indicated having attained advanced competence level and those who were rated as “master” with a score of 5 indicated those that exceeded all expectation in a positive direction. Consequently the total maximum score for the PBL assessment was 65; out of this the weightage of summative assessment for PBL was only 5%.

The PBL assessment rating instrument is being used by the school to evaluate acquisition of PBL skills by the students for more than 25 years. The Centre of Medical Sciences Education (CMSE), UWI, St Augustine reviewed the rating scales and criteria used to assess PBL process by other pioneer medical schools worldwide (such as McMaster University, Canada; Queen’s University, Australia; University of New Mexico, USA; National Autonomous University of Mexico; the University of Malay, Malaysia) and found that the rating scale and criteria used at UWI is quite comparable and comprehensive [8]. An in-house evaluation in 2009 found that 73% of the facilitators found the instrument to be acceptable, user-friendly and it successfully measured the criteria of PBL delivery and assessment [8].

### Ethical approval

Ethical approval for the study was not sought as it was a part of the quality assurance review of the curriculum mandated by the university. It was approved by the Office of the Deputy Dean, Basic Health Sciences, Faculty of Medical Sciences, University of West Indies (UWI), St Augustine Campus, Trinidad and Tobago. The aim of the research was explained to the PBL tutors and they gave their verbal consent to use the PBL ratings in this study. To avoid the disclosure of the personal information of the tutors, the data was codified by the Assessment Unit, Deputy Dean Office.

### Statistical analysis

All calculations and statistics were explored using the Statistical Package for the Social Sciences (SPSS) software Version 21. With a population mean = 50.55 ± 8.20, those tutors’ rating fall below the Z-score of − 1.20 are treated as stringent and above the Z-score of 1.20 are considered to be lenient as presented in Table 1.

**Table 1:** Tutor Mean Ratings Converted to Z-scores

Tutor	M ± SD	Z scores
T_13_	31 ± 3.67	−2.38^a^
T_16_	38.69 ± 6.25	−1.45^a^
T_17_	40.67 ± 3.96	−1.20^a^
T_12_	45.19 ± 11.15	−0.65
T_5_	45.74 ± 5.67	−0.59
T_3_	48.53 ± 7.52	−0.25
T_4_	50.23 ± 4.56	−0.04
T_18_	50.83 ± 4.73	0.03
T_7_	50.96 ± 3.79	0.05
T_6_	51.06 ± 2.64	0.06
T_15_	51.69 ± 0.85	0.14
T_11_	52.76 ± 8.43	0.27
T_8_	54.97 ± 2.57	0.54
T_1_	54.97 ± 6.05	0.54
T_9_	57.73 ± 2.66	0.88
T_14_	60.36 ± 0.93	1.20^a^
T_10_	61.41 ± 2.23	1.32^a^
T_2_	63.03 ± 2.17	1.52^a^

^a^This conversion was done with a population Mean rating of 50.55 and SD of 8.20. Those tutors’ rating fall below the Z-scores of −1.20 are treated as stringent and above the Z-score of 1.20 are considered to be lenient

To find out the significant differences between most lenient versus most stringent raters and highest versus lowest rated groups, independent sample t-test was used. After identifying highest and lowest rated groups; one-way ANOVA followed by post-hoc Bonferroni test was performed to find out the significant effect of tutors in the selected highest and lowest rated groups. Intra class correlation was calculated to determine inter-rater agreements and Pearson product moment correlation was used to find out association between PBL experiences and mean rating of tutors.

## Results

The PBL experience of tutors ranged from 5 to 25 years (mean 12.8 years). The correlation between tutors’ PBL experiences and their mean ratings was found to be moderately significant (*r* = 0.52; *p* < 0.05). The mean rating of male (mean = 51.41 ± 9.44) versus female (mean = 48.83 ± 5.24) was also found to be statistically insignificant ((t-ratio = 0.62; *p* > 0.05).

**Fig. 1:**
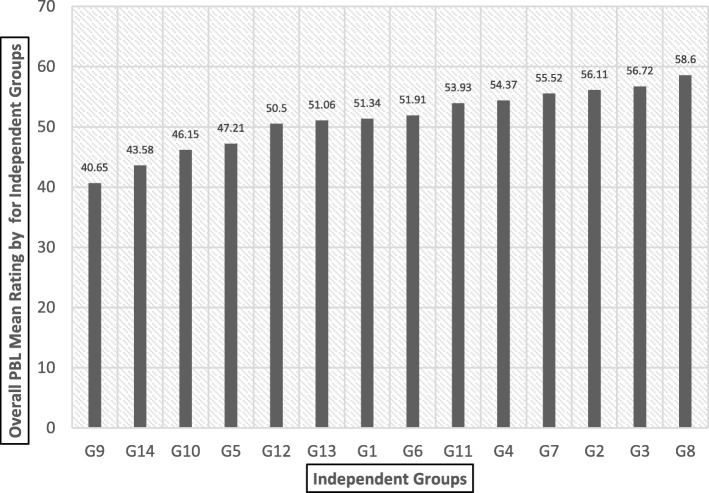
Overall mean ratings for independent groups (G1-G14) in increasing order

**Fig. 2:**
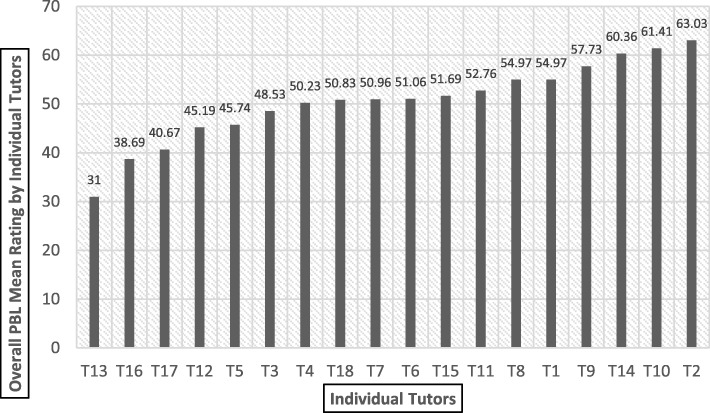
Overall mean rating of individual tutors (T1-T18) in increasing order

The overall mean ratings for each group (G1 through G14) and for each tutor (T1 through T18) was calculated and presented in Fig. 1 and Fig. 2 respectively. Figure 1 shows the mean ratings of all 14 PBL tutorial groups. Further t-ratio reveals that there is a statistically significant difference between highest and lowest rated groups G8 vs. G9 (t-ratio = 12.64; *p* < 0.05).

Figure 2 shows the overall mean rating of individual tutor. The t-ratio reveals there is a statistically significant difference between most lenient and most stringent raters i.e. T2 vs. T13 (t-ratio = 27.96, *p* < 0.05).

Outcome of the one-way ANOVA revealed significant (*p* < 0.01) effect of lenient and stringent tutors for the highest rated group i.e. Group 8 (F = 20.64, with df 2/39) and the lowest rated group i.e. Group 9 (F = 26.00, with df 2/36). In the Table 1, further post-hoc Bonferroni analysis revealed the significant differences (*p* < 0.05) between the tutors in their rating for the highest and lowest rated groups. It was also found that presence of T_10_ (second most lenient tutors - Fig. 2) and T_13_ (the most stringent rating tutor - Fig. 2) might have significantly affected the outcomes. Thus, it can be inferred that the most lenient rating tutor is significantly contributing in enhancing scores of the highest rated group and vice versa.

The intra class correlations (ICC) among rating of different tutors for different groups showed a low agreement among various ratings except three groups (6, 8 and 13) (*r* = 0.40) (Table 2).

**Table 2:** Post-hoc Bonferroni analysis for Highest and Lowest Rated Groups

Multiple Comparisons							
		(J)	Mean Difference (I-J)	Std. Error	Sig.	95% Confidence Interval	
						Lower Bound	Upper Bound
Highest Rated Group:8	Tutor10 61.41 ± 2.23	Tutor11	7.34286^a^	1.15	.000	4.4742	10.2115
		Tutor9	3.14286^a^	1.15	.028	.2742	6.0115
	Tutor11 (52.76 ± 8.43)	Tutor10	−7.34286^a^	1.15	.000	−10.2115	−4.4742
		Tutor9	−4.20000^a^	1.15	.002	−7.0686	−1.3314
	Tutor9 57.73 ± 2.66	Tutor10	−3.14286^a^	1.15	.028	−6.0115	−.2742
		Tutor11	4.20000^a^	1.15	.002	1.3314	7.0686
Lowest Rated Group:9	Tutor12 41.31 ± 10.54	Tutor 13	10.30769^a^	2.59	.001	3.8131	16.8023
		Tutor6	−8.30769^a^	2.59	.008	−14.8023	−1.8131
	Tutor13 31.00 ± 3.67	Tutor12	−10.30769^a^	2.59	.001	−16.8023	−3.8131
		Tutor6	−18.61538^a^	2.59	.000	−25.1100	−12.1208
	Tutor6 49.62 ± 2.43	Tutor12	8.30769^a^	2.59	.008	1.8131	14.8023
		Tutor13	18.61538^a^	2.59	.000	12.1208	25.1100

^a^The mean difference is significant at the 0.05 level

## Discussion

The key findings of the present study are as follows: (i) significant difference between highest and lowest rated groups (t-ratio = 12.64), (ii) significant differences between lenient and stringent tutor’ ratings (t-ratio = 27.96), (iii) Lenient tutors had a significant effect on increasing the group mean scores (F = 20.64), (iv) stringent tutors had a significant effect on decreasing the group mean scores (F = 26.00), (v) disagreement existed among tutor ratings of different groups (*r* = 0.40), and (vi) a significant relationship existed between tutors’ PBL experiences and their mean ratings (*r* = 0.52).

The mean average score rating by the tutors shows that there is a significant difference between the mean rating of highest rater/lenient rater (M = 63.03 ± 2.17) and lowest rater/stringent rater (M = 31.00 ± 3.67). Analysis of lowest rated groups shows that the stringent rater has a significant role in lowering the mean rating of the lowest rated groups (‘dilution effect’) (Table 3)

**Table 3:** The intra class correlations (ICC) showing tutor ratings for different groups

PBL Groups	ICC	95% Confidence Interval	
		Lower Bound	Upper Bound
Group 1	0.109	−0.081	0.425
Group 2	0.004	−0.094	0.234
Group 3	0.282	−0.071	0.663
Group 4	0.120	−0.228	0.547
Group 5	0.176	− 0.178	0.576
Group 6	0.720	0.318	0.902
Group 7	0.123	−0.061	0.441
Group 8	0.412	−0.138	0.781
Group 9	0.159	−0.223	0.600
Group 10	−0.112	−0.181	0.216
Group 11	0.239	−0.118	0.628
Group 12	−0.130	−0.90	0.562
Group 13	0.500	−0.190	0.836
Group 14	0.106	−0.077	0.415

. Further, the lenient rating tutors significantly contributed towards highest mean rating of the tutorial groups. As a matter of leniency, those students who didn’t deserve pass/higher marks got high marks; and because of stringency, those students who deserve higher score, got lower scores. Thus, this puts the good students in disadvantageous situations and vice versa. In analyzing the MRCP(UK) clinical examination (PACES) using multi-facet Rasch modelling, McManus et al. [15] found examiner bias and stringency-leniency-effect have substantial effects on the students’ outcome in clinical examinations. We also found moderately significant correlation between tutors’ PBL experiences and their mean ratings. Previous studies showed that there may be differences in assessment based on tutor experiences [31]. Other factors affecting the assessment of professional skills in PBL included lack of effective interaction, lack of thoroughness, failure to confront students, lack of effort to find solutions, lack of motivation [20]. Research was also focused to explore self-, peer-, and tutor assessment of performance in PBL tutorials among medical students in problem-based learning curricula. It was found that tutor assessment correlated poorly with self-assessment ratings and peer scores correlated moderately with tutor ratings [11, 32].

The present study focused on process assessment of PBL using a locally developed and validated instrument. Process-oriented assessment in PBL focuses on students’ performance during prolonged interactions, which allows the tutors to make a more accurate estimate of a student’s competence when compared with formal examinations [11]. A number of process-oriented instruments were developed by many academic institutes and used to assess the development of PBL skills. Though these instruments are essential to examine PBL skills, they possess psychometric shortcomings which limit their use in high-stake examinations [33, 34]. The University of Maastricht has avoided the use of tutor-based assessment [35], because the dual roles of PBL tutors (i.e. tutor–rater and tutor–teacher) were viewed to be incompatible [35–37]. Literature review showed that the leniency and stringency of PBL tutor ratings in medical schools were not studied widely. Hebert and Bravo [38] used a testing instrument at the Université de Sherbrooke Faculty of Medicine, Canada; their results showed a good correlation of scores with the tutor’s global evaluation (*r* = 0.64). The Newcastle University developed a Group Task exam for summative assessment of students, in which tutors observed a group of students; however, the authors did not report any reliability and validity data [39]. In a study conducted by Dodds et al. (2001), 74 tutors assessed 187 students twice (formative assessment in mid-semester, summative assessment at the end of semester) and tutor scores correlated moderately and significantly with other assessment modalities of each course examined [4]. The authors concluded that scores given by PBL tutors ‘contribute useful, distinctive dimensions to assessment’ in a PBL curriculum. Thus, tutor rating is found to be a valid and reliable form of PBL process assessment. The present study also recorded a disagreement among tutor ratings of different groups (*r* = 0.40), and a significant relationship between tutors’ PBL experiences and their mean ratings (*r* = 0.52).

PBL tutors are important elements in the success of PBL tutorials. It is established that different dimensions of tutor performance influences student learning [40]. In PBL, the role of a tutor is to scaffold student learning which is different from that of teachers in a more traditional medical programme [40–42]. The required tutor activities and commitments in PBL sometimes poses challenges and confusion regarding the tutor’s role in handling learning and students’ ratings [40]. Faculty development and student orientation programmes organized by the medical schools may improve the consistency of scoring and outcomes of the PBL curriculum [40–42]. In our context, robust faculty development may minimize the effect of individual differences of tutor rating.

This study had a small sample size and was performed at a single-center, therefore, caution needs to be taken to generalize the data to other settings. Further studies could be conducted utilizing tutor, peer and self-assessments to examine the reliability of interrater and inter-rater ratings in PBL.

## Conclusion

Ensuring objectivity and maintaining reliability are necessary conditions in order to consider any form of assessment valid. Leniency and stringency factors in the raters affect objectivity and reliability to a great extent as demonstrated in the present study. Thus, more rigorous training in the areas of principles of assessment for the tutors are recommended. Moreover, putting those knowledge and principles to overcome the leniency and stringency subjective factors are essential. Further studies could be conducted triangulating tutor, peer and self-assessment of the PBL process that would also address the effects of any other existing confounding variables such as PBL contents, and difficulty and quality on potential scores. Necessary training is also required to raise the awareness of inevitability of differences of rating which needs to be considered by the tutors while assessing the PBL process.

## Abbreviations

ANOVA: Analysis of Variance
CMSE: Centre for Medical Sciences Education
ICC: Intra class correlations
MBBS: Bachelor of Medicine and Bachelor Surgery
MRCP: Membership of Royal Colleges of Physicians
PACES: Practical Assessment of Clinical Examination Skills
PBL: Problem based learning
SPSS: Statistical Package for the Social Sciences
UWI: The University of The West Indies
